# Single Nucleotide Polymorphisms in *CDKAL1* Gene Are Associated with Risk of Gestational Diabetes Mellitus in Chinese Population

**DOI:** 10.1155/2019/3618103

**Published:** 2019-04-14

**Authors:** Keke Wang, Qiong Chen, Yongliang Feng, Hailan Yang, Weiwei Wu, Ping Zhang, Ying Wang, Jamie Ko, Feng Zhao, Wenqiong Du, Feifei Yang, Tianbi Han, Suping Wang, Yawei Zhang

**Affiliations:** ^1^Department of Epidemiology, Shanxi Medical University School of Public Health, Taiyuan 030001, China; ^2^Office for Cancer Prevention and Research, Affiliated Cancer Hospital of Zhengzhou University/Henan Cancer Hospital, Zhengzhou 450008, China; ^3^Department of Obstetrics, the First Affiliated Hospital, Shanxi Medical University, Taiyuan 030001, China; ^4^Department of Chronic Disease Epidemiology, Yale School of Public Health, New Haven, 06520 CT, USA; ^5^Department of Surgery, Yale University School of Medicine, New Haven 06520, USA; ^6^Department of Environmental Health Sciences, Yale School of Public Health, New Haven, 06520 CT, USA

## Abstract

Gestational diabetes mellitus (GDM) is a growing public health concern for many reasons, and its etiology remains unclear. Due to the similarity of its pathophysiology with type 2 diabetes (T2DM), we evaluated the relationship between published T2DM susceptibility genes and the risk of GDM. A total of 303 SNPs from genes including *IRS1*, *IGF2BP2*, *CDKAL1*, *GCK*, *TCF7L2*, *KCNQ1*, and *KCNJ11* and the risk of GDM were examined in a nested case-control study with 321 GDM cases and 316 controls. The odds ratios (ORs) and their 95% confidence interval (95% CI) were estimated by unconditional logistical regression as a measure of the associations between genotypes and GDM in additive, recessive, dominant, and codominant models adjusting for maternal age, maternal BMI, parity, and family history of diabetes. At the gene level, *CDKAL1* was associated with GDM risk. SNPs in the *CDKAL1* gene including rs4712527, rs7748720, rs9350276, and rs6938256 were associated with reduced GDM risk. However, SNPs including rs9295478, rs6935599, and rs7747752 were associated with elevated GDM risk. After adjusting for multiple comparisons, rs9295478 and rs6935599 were still significant across the additive, recessive, and codominant models; rs7748720 and rs6938256 were significant in dominant and codominant models; and rs4712527 was only significant in the codominant model. Our study provides evidence for an association between the *CDKAL1* gene and risk of GDM. However, its role in the GDM pathogenesis still needs to be verified by further studies.

## 1. Introduction

Gestational diabetes mellitus (GDM) is a public health concern due to its large disease burden and its short- and long-term adverse health impact on pregnant women and their offspring including obesity, metabolic syndrome, and type 2 diabetes mellitus (T2DM) [1]. GDM affects approximately 5-17% of all pregnancies worldwide [2]. The prevalence has increased over the past 20 years, and this upward trend is expected to continue due to a rising number of overweight or obese women of childbearing age [2, 3]. Although the pathogenesis of GDM remains unclear, it is considered as a complex disease caused by multiple factors including genetic, environment, lifestyle, and other random factors that related to reduction of insulin sensitivity and insulin resistance.

GDM shares common risk factors with T2DM, including family history of diabetes, obesity, high maternal age, abnormal glucose tolerance, and specific ethnicity [4]. The similarities of pathophysiology between GDM and T2DM may suggest that the genetic factors of T2DM may also be involved in the development of GDM. GDM has been suggested as precursor for T2DM by some researchers [5]. Genome-wide association studies and candidate gene studies had already identified several genes that associated with risk of T2DM, and some of them were also verified to be associated with GDM risk by candidate gene studies and one GWA study including *TCF7L2*, *GCK*, *KCNJ11*, *KCNQ1*, *CDKAL1*, *IGF2BP2*, and *IRS1* genes [6]. However, there are still many genes that were previously reported to be associated with T2DM but were not associated with GDM risk. It is possible that the T2DM susceptibility genes were mostly found in GWA studies or meta-analysis normally with large sample size and weak effect, but those found in GDM studies often have small sample size resulting in insufficient statistical power to identify weak effects [7].

Current evidence suggested that the genetic polymorphisms often had different GDM risks in different ethnic populations [5, 8]. Genetic polymorphisms in *TCF7L2* and *GCKR* genes were found to be associated with GDM risk in European women, and polymorphisms in *TSPAN8* were associated with increased GDM risk in African American women [9]. *TCF7L2* and *KCNQ1* polymorphisms were found to be associated with GDM risk in Mexican women [10]. In Asia, polymorphisms in *TCF7L2*, *KCNQ1*, *KCNJ11*, *IGF2BP2*, and *CDKAL1* genes were associated with GDM risk in the Korean population [8], while *CDKAL1* and *GCK* gene polymorphisms were associated with GDM in Indian and Thai women [8]. However, to our knowledge, the association with GDM risk was only evaluated in *IGF2BP2* and *KCNQ1* genes in Chinese women [7], and other genes like *TCF7L2*, *GCK*, *KCNJ11*, *CDKAL1*, and *IRS1* still need to be verified by additional studies. Thus, we examined the associations between these genes and risk of GDM using a nested case-control study design in the Taiyuan birth cohort population.

## 2. Materials and Methods

### 2.1. Subjects

The subjects were enrolled from an ongoing birth cohort at the First Affiliated Hospital of Shanxi Medical University during March 1, 2012, and July 30, 2014, in Taiyuan, China [11]. Eligible women included pregnant women who came to the hospital for delivery with gestational age of 20 weeks or more, who had no mental illness, and who were aged 18 years or older. Eligible women were informed of the study procedure upon their arrival at the hospital for delivery. After obtaining written consent, an in-person interview was conducted at the hospital by trained study interviewers using a standardized and structured questionnaire. The questionnaire collected information regarding demographic factors, reproductive and medical history, smoking, alcohol and tea consumption, occupational and residential histories, physical activity, and diet. Information on birth outcomes and pregnancy complications was abstracted from medical records.

### 2.2. Case and Control Selection

Blood glucose was tested using a 75 g oral glucose tolerance test (OGTT) during 24-28 weeks of gestation. Subjects were diagnosed as having GDM if they met at least one of the following criteria: (1) fasting blood glucose > 5.1 mmol/L, (2) 1-hour blood glucose > 10.0 mmol/L, and/or (3) 2-hour blood glucose > 8.5 mmol/L. A total of 334 women had GDM (cases), and 334 control subjects who had no GDM were randomly selected through frequency matching to cases by age, month of conception, and residence. 13 cases and 18 controls were excluded due to missing genotyping per person or per SNP. Finally, 321 cases and 316 controls were included in the analysis.

### 2.3. Genotyping

DNA was extracted, isolated, and purified from whole blood samples according to a standard phenol-chloroform extraction method. Genotyping was conducted using an Illumina GoldenGate Platform. Duplicate samples (5%) were interspersed throughout the plates used for genotype analysis for quality control purposes. A total of 303 single-nucleotide polymorphisms (SNPs) from genes including *IRS1*, *IGF2BP2*, *CDKAL1*, *GCK*, *TCF7L2*, *KCNQ1*, and *KCNJ11* were considered for this study. The completion rate for all SNPs was over 99%. The Hardy-Weinberg equilibrium (HWE) was assessed in controls for each SNP using a chi-square test. SNPs with a *P* value >0.05 from the chi-square test were considered to be in HWE. Of the 303 SNPs tested, 8 SNPs were not in HWE and were excluded from the final analysis (as shown in Supplementary Table 1).

### 2.4. Statistical Analysis

All statistical analyses were carried out using R statistical software version 3.3.1. The odds ratios (OR) and their 95% confidence intervals (95% CI) were estimated by unconditional logistical regression as a measure of the associations between genotypes and GDM adjusting for maternal age, maternal BMI, parity, and family history of diabetes. The associations were estimated in additive, dominant, codominant, and recessive models. The minimum *P* (min*P*) tests were conducted to examine an association at the gene level using “minPtest” R package [12]. The min*P* test was based on permutation resampling and was conducted to assess the true statistical significance of the smallest *P* trend within each gene region. Haplotype analyses were conducted for all genes in which more than 1 SNP was genotyped. Haplotype block structures were evaluated with HaploView version 4 using the method of Four Gamete Rule. Logistic regression was used to calculate the ORs of the haplotypes using the method implemented in the “haplo.ccs” package [13]. Only major haplotypes with an estimated frequency of over 5% are considered in this report. The ORs were computed compared to haplotype with the highest estimated population frequency. An additive model was tested adjusting for maternal age, maternal BMI, parity, and family history of diabetes. The false discovery rate (FDR) method with a significance level of 0.2 was applied for multiple comparisons. Gene-by-gene interactions were detected using the method of generalized multivariate dimensionality reduction (GMDR) [14].

### 2.5. Results

The characteristics of the cases and controls have already been described elsewhere [11]. In brief, there were more GDM cases with a higher BMI and family history of diabetes than control subjects (*P* = 0.0002, *P* = 0.005). None of the cases or controls consumed alcohol during pregnancy. The distributions of maternal age, weight gain during pregnancy, parity, high blood pressure during pregnancy, passive smoking, and gestational weeks between cases and controls were similar.

All associations between genotypes in the seven genes and GDM risk are shown in Supplementary Table 2. Significant associations with GDM risk in the *CDKAL1* gene are shown in Table 1; risk alleles of A, G, and G in rs9295478, rs6935599, and rs7747752 were found to be associated with elevated GDM risk in additive, dominant, codominant, and recessive models. These alleles could increase the GDM risk for around 2 times in the additive model, 1.45 times in the dominant model, 1.4 times in the codominant model, and 1.8 times in the recessive model. These associations remained statistically significant after adjusting for multiple comparisons in additive, recessive, and codominant models for rs9295478 and rs6935599.

G alleles in rs4712527, rs7748720, rs9350276, and rs6938256 were found to be associated with reduced GDM risk compared with A alleles in all four models as mentioned above. GDM risks in women who carried G alleles in rs4712527 and rs7748720 were approximately 0.20 times in the additive model, 0.50 times in dominant and codominant models, and 0.20 times in the recessive model compared with women who carried A alleles. GDM risks in women who carried G alleles in rs9350276 and rs6938256 were around 0.40 times in the additive model, 0.60 times in dominant and codominant models, and 0.50 times in the recessive model compared with women who carried A alleles. After adjusting for multiple comparisons, the associations for rs7748720 and rs6938256 remained significant in dominant and codominant models, and rs4712527 remained significant in the codominant model; however, the nonsignificant results were found in rs9350276.

As shown in Table 2, *CDKAL1* gene polymorphisms were significantly associated with risk of GDM at the gene level, and this gene was still statistically significant after adjusting for multiple comparisons. Nonsignificant results were found in *IRS1*, *IGF2BP2*, *GCK*, *TCF7L2*, *KCNQ1*, and *KCNJ11* genes.

**Figure 1:**
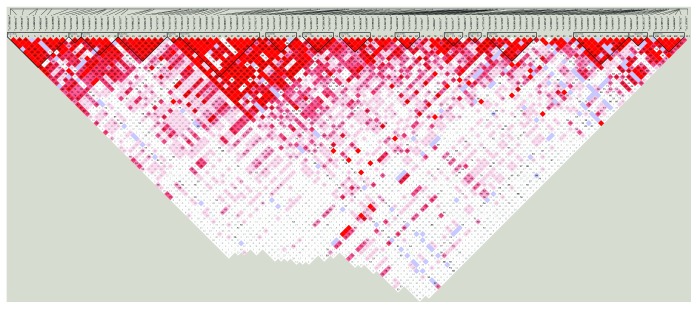
LD plot of 111 SNPs in the *CDKAL1* gene.

We observed strong linkage disequilibrium of 16 blocks in 111 SNPs in the *CDKAL1* gene (Figure 1). SNPs significantly associated with GDM that are listed in Table 1 in the manuscript including negative SNPs (rs4712527 and rs7748720) and positive SNPs (rs9295478, rs6935599, and rs7747752) belong to the same block 4, another SNP rs9350276 belong to block 5, and rs6938256 does not belong to any block constructed by HaploView. The GACGGACG haplotype in block 4 and the AG haplotype in block 5 were associated with reduced risk of GDM. Haplotype analyses were consistent with the results of the individual SNP analyses and did not provide additional insight into these associations (Table 3). Gene and gene interaction analysis using GMDR did not find significant results.

## 3. Discussion

We verified associations between polymorphisms in genes including *TCF7L2*, *GCK*, *KCNJ11*, *KCNQ1*, *CDKAL1*, *IGF2BP2*, and *IRS1* and risk of GDM in a Chinese population. At the gene level, *CDKAL1* was significantly associated with GDM risk. The *CDKAL1* gene located in the short arm of human chromosome 6 could affect the function of *β*-cells through inhibiting the activity of *CDK5* and acting as a tRNA-modifying enzyme [15], and risk alleles in this gene could affect the process of proinsulin conversion to insulin through protein translation. In our study, several SNPs in the *CDKAL1* gene were found to be associated with GDM risk. Among these SNPs, the G allele in rs4712527 was reported to be associated with reduced risk of T2DM in a cohort study from Argentina [16], but not in a Chinese population [17]. *CDKAL1* gene polymorphisms including rs7754840 and rs10946398 were reported to be associated with risk of T2DM in Asian populations [18, 19]. Results from a GWA study in Korean women suggested that rs7754840 in *CDKAL1* is strongly associated with GDM [20], and subsequent meta-analysis also confirmed that the C allele of rs7754840 was associated with elevated risk of GDM [6]. A study from India did not find a significant association between *CDKAL1* polymorphisms and GDM risk [21]. Another study from Russia examined whether *CDKAL1* polymorphisms modified the relationship between lifestyles including food consumption, physical activity, and smoking as well as GDM risk and it did not find significant interactions; however, results from this study suggest that the association of sausage consumption with GDM risk can be determined based on the number of risk alleles of rs1799884 in *GCK* [22]. The *CDKAL1* rs10946398 CC genotype was reported to be associated with the need for insulin therapy in a study conducted in Poland; however, the association did not pass the statistical significance threshold after correction for multiple testing [23]. Rs7748720 in *CDKAL1* was found to be significantly associated with GDM risk; however, it was never reported in previous studies, and it still has to be verified by further studies in a different population.

Although the precise pathology of GDM remains unclear, it was reported to be related to increased insulin resistance and/or decreased insulin sensitivity during pregnancy [1]. Therefore, the association between GDM risk and *CDKAL1* polymorphisms could be supported by its relation to these metabolic characteristics. In a Cardiometabolic Risk in Chinese (CRC) study, rs10946398 of *CDKAL1* was associated with markers of impaired insulin secretion, suggesting that its effect on glucose-related traits might have a role in the development of GDM [24]. Among Greek children, rs9356744 and rs2206734 were related to insulin resistance, a clinical marker related to T2DM in a cross-sectional cohort of Greek children and adolescents of European descent [25]. In a meta-analysis, rs9460557 of *CDKAL1* showed evidence with insulin clearance and suggested it may have a pleiotropic effect on insulin secretion, insulin sensitivity, and insulin clearance [26]. rs7756992 was also validated in relation to these intermediate traits related to T2DM in an Indian population [27].

GWA and candidate gene studies have increased our knowledge of loci associated with diabetes; however, applying these findings to infer pathophysiology and promote drug discovery remains challenging. *CDKAL1* was revealed to be an inhibitor of *CDK5* to suppress the *β*-cell differentiation and further highlighted its importance in diabetes [28]. T2DM-related alleles of SNPs located within the *CDKAL1* gene region were reported to be associated with impaired *β*-cell function [29] and impaired insulin secretion [24, 30], which may suggest the role of *CDKAL1* in the development of GDM. Mutation in *CDKAL1* led to impaired glucose secretion in vitro and vivo [31], and a high-content chemical screen identified a candidate drug that rescued *CDKAL1*-specific defects by inhibiting the FOS/JUN pathway [31]. This may help to the precise therapy of diabetes.

Strengths and limitations should be considered when interpreting the study results. Firstly, diagnosis of GDM in our study was acquired by investigating the well-documented medical records, which minimized potential disease misclassification. Secondly, information on potential confounders was collected using a standardized and structured questionnaire allowing for the control of potential confounding effects. The current study was hospital-based, which might have limited the generalizability of these study results. Thirdly, the BMI was not equally distributed between cases and control subjects; however, it was adjusted in each model analyzed. The results in our study will need to be verified by larger population-based studies. Lastly, the sample size in our study was moderate, and the weak effects of SNPs on GDM may not have been observed.

To the best of our knowledge, our study for the first time found a novel association between rs7748720 of *CDKAL1* and GDM risk. This would help increase our knowledge of loci associated with GDM and infer the pathophysiology; however, additional studies still need to verify the results.

## Figures and Tables

**Table 1:** SNPs in CDKAL1 gene and risk of gestational diabetes mellitus^#^.

SNPs	Genotypes	Cases	Controls	Additive		Dominant		Recessive		Codominant	
				OR (95% CI)	*P*	OR (95% CI)	*P*	OR (95% CI)	*P*	OR (95% CI)	*P*
rs4712527	AA	245	208	0.21 (0.05-0.69)	0.019^∗^	0.59 (0.41-0.84)	0.003^∗^	0.24 (0.05-0.77)	0.029	0.59 (0.43-0.81)	0.001^∗∗^
	AG	73	96								
	GG	3	12								

*rs7748720*	AA	241	193	0.15 (0.03-0.47)	0.003^∗^	0.51 (0.36-0.72)	0.0002^∗∗^	0.18 (0.04-0.55)	0.007	0.51 (0.37-0.70)	0.00003^∗∗^
	AG	75	107								
	GG	3	15								

rs9295478	GG	72	93	2.08 (1.33-3.27)	0.001^∗^	1.46 (1.01-2.10)	0.042	1.83 (1.26-2.66)	0.001^∗^	1.44 (1.15-1.81)	0.002^∗∗^
	GA	149	160								
	AA	100	63								

rs6935599	AA	72	93	2.05 (1.31-3.22)	0.002^∗^	1.45 (1.01-2.10)	0.044	1.80 (1.24-2.62)	0.002^∗^	1.43 (1.14-1.79)	0.002^∗∗^
	AG	149	160								
	GG	99	63								

rs7747752	CC	76	96	1.96 (1.25-3.09)	0.004^∗^	1.45 (1.01-2.08)	0.042	1.69 (1.16-2.48)	0.006^∗^	1.39 (1.11-1.75)	0.004^∗∗^
	CG	153	159								
	GG	92	60								

rs9350276	AA	188	155	0.46 (0.26-0.81)	0.008^∗^	0.67 (0.49-0.92)	0.014	0.52 (0.30-0.90)	0.021	0.70 (0.55-0.89)	0.004^∗∗^
	AG	110	122								
	GG	23	39								

rs6938256	AA	207	165	0.39 (0.20-0.74)	0.005^∗^	0.58 (0.42-0.81)	0.001^∗∗^	0.46 (0.24-0.87)	0.019	0.63 (0.48-0.81)	0.0005^∗∗^
	AG	96	118								
	GG	16	30								

∗ and ∗∗ indicate that the associations were statistically significant at the 0.20 and 0.05 levels after adjusting for multiple comparisons using the FDR method. ^#^ORs were adjusted for maternal age, maternal BMI, parity, and family history of diabetes.

**Table 2:** Gene polymorphisms and risk of gestational diabetes mellitus.

Genes	Location	Start	End	Number of SNPs	min*P*	min*P* ^∗^
IRS1	2q36.3	227,596,033	227,664,485	4	0.362	0.656
IGF2BP2	3q27.2	185,361,527	185,542,844	14	0.515	0.709
CDKAL1	6p22.3	20,534,688	21,232,635	111	*0.001*	*0.007*
GCK	7p13	44,183,870	44,237,769	15	0.157	0.550
TCF7L2	10q25.2	114,710,009	114,927,437	43	0.608	0.709
KCNQ1	11p15.5	2,465,914	2,870,340	107	0.375	0.656
KCNJ11	11p15.1	17,406,795	17,410,878	1	0.923	0.923

min*P* ^∗^ after adjusting for multiple comparisons using the method of FDR.

**Table 3:** Association of haplotypes in the CDKAL1 gene with gestational diabetes mellitus.

SNP combinations	Frequency (%)	OR^a^	95% CI	*P* ^b^
*Block 4*				
rs4712527 rs9356744 rs9368222 rs7748720 rs9295478 rs6935599 rs7747752 rs6928012				
AGAAAGGG	39.88	1		
AACAGACA	31.24	0.82	0.62-1.07	0.145
*GACGGACG*	*14.08*	*0.52*	*0.37-0.74*	*0.0003*
AACAAGGG	6.72	0.98	0.62-1.55	0.931
Rare (<0.05)	8.08	0.86	0.56-1.33	0.505

*Block 5*				
rs13217519 rs9350276				
GA	36.73	1		
AA	35.32	1.03	0.79-1.33	0.845
*AG*	*27.94*	*0.71*	*0.54-0.93*	*0.015*

## Data Availability

The data used to support the findings of this study are included within the article and supplementary information files.

## Supplementary Materials

Supplementary Materials Supplementary Table 1: genes and SNPs evaluated. Supplementary Table 2: genotypes and risk of gestational diabetes mellitus.
